# Magma injection beneath the urban area of Naples: a new mechanism for the 2012–2013 volcanic unrest at Campi Flegrei caldera

**DOI:** 10.1038/srep13100

**Published:** 2015-08-17

**Authors:** Luca D’Auria, Susi Pepe, Raffaele Castaldo, Flora Giudicepietro, Giovanni Macedonio, Patrizia Ricciolino, Pietro Tizzani, Francesco Casu, Riccardo Lanari, Mariarosaria Manzo, Marcello Martini, Eugenio Sansosti, Ivana Zinno

**Affiliations:** 1 Istituto Nazionale di Geofisica e Vulcanologia, sezione di Napoli Osservatorio Vesuviano, via Diocleziano 328, 80124 Napoli Italy; 2 National Research Council of Italy (CNR), Istituto per il Rilevamento Elettromagnetico dell’Ambiente, via Diocleziano 328, 80124 Napoli Italy

## Abstract

We found the first evidence, in the last 30 years, of a renewed magmatic activity at Campi Flegrei caldera from January 2012 to June 2013. The ground deformation, observed through satellite interferometry and GPS measurements, have been interpreted as the effect of the intrusion at shallow depth (3090 ± 138 m) of 0.0042 ± 0.0002 km^3^ of magma within a sill. This interrupts about 28 years of dominant hydrothermal activity and occurs in the context of an unrest phase which began in 2005 and within a more general ground uplift that goes on since 1950. This discovery has implications on the evaluation of the volcanic risk and in the volcanic surveillance of this densely populated area.

Understanding the unrest dynamics in calderas is still an open scientific issue. Geophysical and geochemical signals often show puzzling patterns that make the correct interpretation of volcanic unrests a difficult task. This problem has important implications in the management of volcanic risk since calderas are generally densely populated because of their relatively flat topography and fertile soil. A particularly relevant scientific problem is to discriminate whether caldera unrests are related to perturbations of a shallow hydrothermal system or driven by genuine magmatic intrusions^1^.

Here we study the case of Campi Flegrei caldera (CFc), one of the areas with the highest volcanic risk in the world since it hosts a large part of the Naples metropolitan area (Italy), thus threatening about 1.5 million people (Fig. 1)^2^.

The current CFc landscape has been shaped by two major caldera-forming eruptions: the Campanian Ignimbrite (CI, 40 ky) with Volcanic Explosivity Index (VEI) of 7 and the Neapolitan Yellow Tuff (NYT, 15 ky) with VEI = 6^3^. After the NYT eruption, CFc experienced tens of minor eruptions, with VEI ranging from 0 to 5^3^,^4^. The last eruption (Monte Nuovo, VEI = 2) occurred in 1538 and was preceded by significant ground uplift and earthquakes, starting at least 70 years before the eruption^5^ (Fig. 1A). During the XX century, CFc underwent three episodes of major ground uplift: 1950–1952 (0.73 m), 1969–1972 (1.77 m) and 1982–1984 (1.79 m)^6^. More than 16000 earthquakes accompanied the latter episode with magnitudes up to 4^7^. This led to the temporary evacuation of about 40000 people from the town of Pozzuoli, for several months^8^. Various authors inferred a magmatic source for this crisis^9^,^10^.

After two decades of prevailing subsidence, since 2005 the uplift at CFc resumed, showing a highly unsteady behavior with intervals of increased uplift rate, alternating with intervals of subsidence or stationary deformation trends^7^,^11^. In very recent years, CFc has experienced an accelerating ground uplift rate: during the April 2012—January 2013 time interval, the caldera has shown a rapid uplift of about 11 cm with a peak rate of about 3 cm/month during December 2012^11^,^12^ (Fig. 1B); this event led the Italian Civil Protection to raise the alert level of the volcano from “background” to “attention”.

In recent years (1985–2011), the dynamics of CFc has been mostly linked to its hydrothermal system^13^,^14^,^15^,^16^. Chiodini *et al.* [2015] also showed a remarkable correlation between ground deformation and geochemical parameters in the interval 2005–2011: they interpreted this correlation as the “signature” of transient disturbances propagating through the hydrothermal system. However, this correlation broke down in 2012, suggesting that the driving mechanism of the ground uplift changed. We found strong evidences that the recent dynamics of CFc can be explained as the emplacement of a magma batch within a flat, sill-shaped, magmatic reservoir.

## Results

### Source kinematics

**Figure 1:**
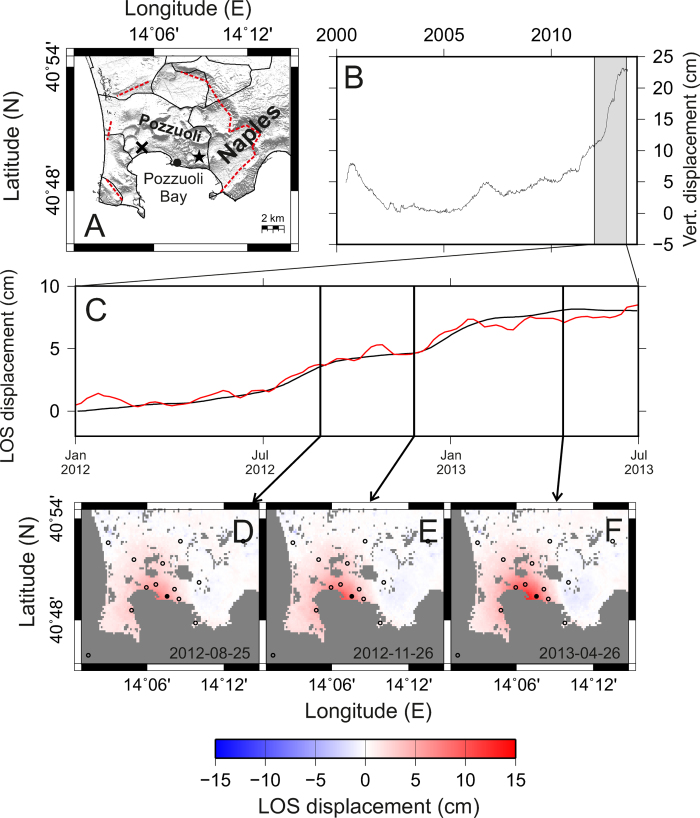
GPS and DInSAR results. (**A**) Shaded relief map of the Campi Flegrei area. Red dashed lines mark the approximate boundaries of the caldera. The star is the position of the Solfatara crater while the cross is the location of the M. Nuovo (1538 AD) eruptive vent. The black circle is the position of the GPS station RITE. (**B**) Vertical component of GPS station RITE measurements for the interval 2000–2014 (the shaded area indicates the analyzed interval). (**C**) Comparison between the GPS displacement projected along the satellite Line-of-Sight (black line) and the DInSAR time series (red line). (**D**–**F**) DInSAR deformation maps in three selected intervals. Black circles indicate the position of GPS stations used in the geodetic imaging. All the maps were realized with the software GMT (http://gmt.soest.hawaii.edu).

In our study we investigated the 2012–2013 ground uplift episode by using a multiparametric dataset. We exploited the Differential SAR Interferometry (DInSAR) technique^17^,^18^ which has proven to be an effective tool for studying ground deformation at calderas^19^,^20^,^21^. For our purposes, we investigated a dataset acquired by the COSMO-SkyMed (CSK) satellite constellation in order to produce ground deformation time series of the CFc area, through SBAS-DInSAR approach^17^ (Fig. 1C–F). This dataset has been complemented by measurements of 14 continuous GPS stations, belonging to the INGV-OV permanent monitoring network (Fig. 1C)^11^ (see “Processing of DInSAR and GPS data” in the Methods section).

The major ground uplift episode which occurred during 1982–1984 has been attributed, by several authors, to a sill-like planar structure source^10^,^22^,^23^. For what concerns the interval 2012–2013, Trasatti *et al.* (2015) have shown that, using a moment-tensor point source, the best fit result is consistent with a sill-shaped magma body. In this work we investigated the same time interval but we inferred about the full source kinematics by using a finite time-varying model and we exploited a physical model of the emplacement of magma within the sill.

**Figure 2:**
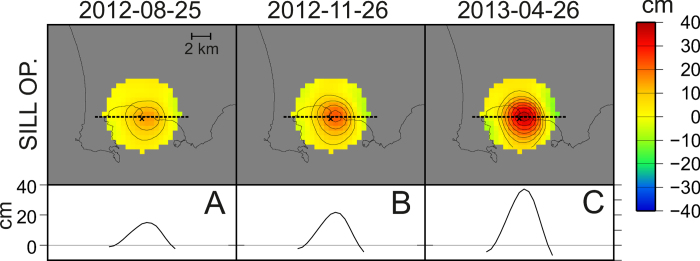
Results of the geodetic imaging for three selected intervals. (**D**–**F** in Fig. 1). The cross represents the point of coordinates (14.20°E 40.82°N). (**A**–**C**) Opening of the sill (at 3100 m depth). On the top of each panel we show maps of the sill opening. Contours spacing is 5 cm. On the bottom we represent the opening along the profiles marked by dashed lines. All the maps were realized with the software GMT (http://gmt.soest.hawaii.edu).

Accordingly, we first determined the most likely depth of such a possible source for the period 2012–2013, using a non-linear optimization procedure based on our ground deformation dataset (DInSAR and GPS). The results showed that the source is located at a depth of 3090 ± 138 m b.s.l. (see “Non-linear optimization” in the Methods section). This depth is fairly consistent with that of previous works using finite planar source models at Campi Flegrei^10^,^22^,^23^. Once the source depth was fixed, we applied a geodetic imaging technique to reconstruct the kinematics of the sill intrusion process (see “Geodetic imaging” in the Methods section). The retrieved spatial and temporal pattern of the source geometry (Fig. 2) is compatible with a growing sill that, at the end of the considered period, exhibits a slightly elliptical geometry with an extension of about 5 km towards NNW-SSE and about 4.5 km towards ENE-WSW. The retrieved maximum aperture of the sill is of about 35 cm at its center (Fig. 2C).

### Determination of the physical parameters of the sill

**Figure 3:**
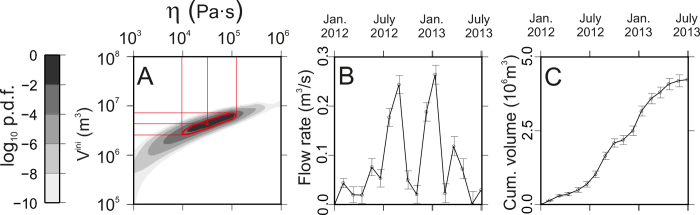
Model of the dynamics of the sill intrusion. (**A**) “a posteriori” p.d.f. as a function of magma viscosity (η) and of the amount of liquid magma already present in the sill before the 2012–2013 episode (V^ini^). The red contour encloses the area with 95% of probability. The corresponding confidence bounds are delimited by the red lines. (**B**) Inferred magma flow rate. (**C**) Cumulative volume (Cum. volume) of injected magma.

In order to determine the physical properties of the injected magma and the time-varying injection rate (see “Inversion of the sill parameters” in the Methods section), we applied the numerical model of sill intrusion developed in reference^24^ that exploits the kinematics retrieved from the geodetic imaging. This approach allowed us to infer that the fluid inside the sill has an average viscosity of about 3.1 × 10^4^ Pa·s with confidence bounds ranging from 0.9 × 10^4^ to 1.2 × 10^5^ (Fig. 3A). This range of viscosities is compatible with gas-poor melts, which have the chemistry of the most commonly erupted magmas at CFc in the last millennia^25^. This finding strongly supports our hypothesis about the magmatic nature of the 2012–2013 uplift. Furthermore, the model requires that, before 2012, a small amount of magma was already present inside the sill: 0.004 km^3^ with confidence bounds ranging from 0.0025 to 0.0063 km^3^ (Fig. 3A). Albeit this model does not take into account the contribution to the ground deformation of gases released from the magma (and injected into the hydrothermal system) our approach is still suitable since, in the considered period, the role of the hydrothermal system as a deformation source is limited^26^. Moreover, we note that after 2013 there has been no significant subsidence in Campi Flegrei^11^. This is a further evidence to support the hypothesis of a dominant magmatic origin for the 2012–2013 ground deformation.

The retrieved time-varying injection rate (Fig. 3B) has two main peaks in September and December 2012, and a smaller one in March 2013. The first two peaks have a value of about 0.25 m^3^/s and a duration of 3–4 months. The total amount of injected magma from January 2012 to July 2013 is of about 0.0042 ± 0.0002 km^3^ (Fig. 3C). This value is of the same order of magnitude of the amount of magma already present in the sill and it is compatible with small-size eruptions (VEI = 1–2) such as those occurred in the caldera during the last 15 ky^27^.

### Stress field variations

**Figure 4:**
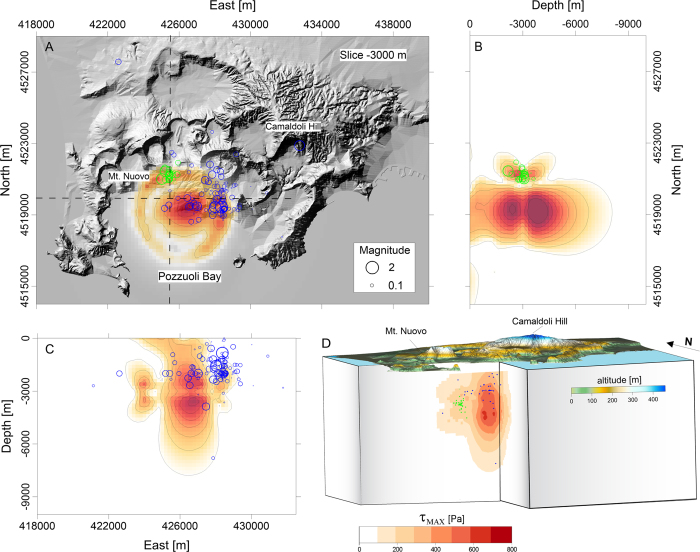
Elastic stress field variations due to the intrusion of the sill. We considered a cumulative opening of 12 cm relevant to January-September 2012 time interval. (**A**) Map of τ_MAX_ (maximum shear stress) at a depth of 3000 m b.s.l. superimposed on the shaded relief map of the CFc. Earthquake hypocenters are reported in green for the September 2012 seismic swarm, while they are in blue for the 2005–2012 interval. In (**B**,**C**) the N-S and E-W cross-sections of τ_MAX_ are reported. (**D**) 3D view of τ_MAX_ field distribution resulting from the structural mechanics modeling. All the maps were realized with the software Surfer®.

The emplacement of a sill also explains anomalies in the recorded seismicity. Indeed, on September 7^th^ 2012 there was a seismic swarm, located beneath the town of Pozzuoli, consisting of about 200 earthquakes (maximum magnitude 1.8) that occurred within an interval of about 1.5 hours. Their hypocenters were located outside the area affected by microearthquakes in the previous years (see Fig. 4, where the green circles represent the hypocenters of this swarm); instead, these earthquakes were located very close to the northern edge of the inferred inflating magmatic reservoir. Stress changes play a fundamental role in the seismicity of calderas^28^. The simultaneous occurrence of this swarm and the first injection rate peak (Fig. 3B) suggests a strong relationship between the two phenomena. To investigate this point, we modeled the stress field variation due to the magmatic intrusion, in the considered time interval, and we found that the modeled source causes a significant increase in the maximum shear stress along the rim of the sill (Fig. 4) (see “Finite Element Modeling of the stress field” in the Methods section).

## Discussion

Our findings suggest that the emplacement of a magmatic sill beneath CFc is the driving mechanism for the 2012–2013 accelerated ground uplift. A similar result was obtained by Trasatti *et al.* (2015), whose estimate of the volumetric variation (about 0.002 km^3^) is compatible with our findings. For what concerns the nature of the source, our fluid-mechanical numerical modeling supports its magmatic origin in terms of viscosity (Fig. 3A).

This also provides a key to interpret the caldera unrest phase that started about 60 years ago and led to a maximum uplift in the area of more than 3 m. In fact, the observed uplift phenomena can be interpreted in terms of intermittent injections of limited magma batches feeding the growth of a shallow magmatic reservoir associated with transient perturbations of the hydrothermal system. The inferred presence of liquid magma before 2012 probably reflects a persistent structure which has been repeatedly refilled in the last decades. Geological evidences of this long-term behavior are present in the abundant layers of sub-volcanic rocks that are found in various boreholes^29^. Moreover, petrological studies of the crystal size distribution in volcanic rocks that at CFc show evidences of small, shallow magmatic chambers, hosting the magma for few months before small-medium size eruptions take place^3^. Therefore, we conclude that repeated emplacement of sills is an important mechanism in both the short and the long-term evolution of CFc^22^. This mechanism implies a transient nature of shallow magma bodies, possibly explaining the substantial lack of detection by typical geophysical prospection techniques^30^,^31^,^32^. This model has also implications on the evaluation of vent opening in calderas since it suggests that the vent opening probability is time varying, depending on the sill radius and the maximum shear stress changes.

This study suggests that a deeper understanding of unrest pattern at CFc requires a change in the reference paradigm for the interpretation of precursors. Conceptual models predicting a monotonic acceleration of the observed parameters should be carefully re-evaluated in these volcanic contexts. The possible presence of extended magmatic bodies at shallow depth within calderas suggests that DInSAR data, together with high-rate, high sensitivity, multiparametric ground based measurements (e.g. borehole seismology, microgravity, tilt, strain, GPS and geochemistry) are needed to track possible rapid and tricky changes in the observed patterns. Accordingly, the observation of volcanic precursory phenomena in the short-term, as well as the development of innovative real-time analysis techniques, should be taken into account for an effective surveillance of Campi Flegrei caldera and, more generally, of other active calderas.

## Methods

### Processing of DInSAR and GPS data

In order to investigate the ground deformation pattern of Campi Flegrei caldera with centimetric precision and high spatial and temporal resolution^18^, we exploited 298 SAR images, acquired from 2009 to 2014 by the COSMO-SkyMed satellites on ascending orbits. We computed 921 differential interferograms with a multilook (spatial averaging) factor of 10 in both directions, thus reducing the final resolution to about 30 m by 30 m^33^; the look angle is of about 44°. Finally, the multilook DInSAR interferograms were inverted through the SBAS-DInSAR algorithm^17^ and the corresponding displacement time-series (projected along the radar line of sight) was retrieved for each investigated coherent pixel. The SAR data processing was performed by considering as reference pixel a stable point located near the Naples harbor. GPS data comes from the permanent INGV-OV Campi Flegrei monitoring network. Details about their processing can be found on De Martino *et al.* (2014)^11^. Both DInSAR and GPS time series have been filtered by using a Gaussian filter with a width of about 30 days and resampled on 100 equally spaced intervals.

### Non-linear optimization

The first step to model the observed ground deformation was fitting the data with a simple time varying model. We adopted the sill model proposed by Fialko *et al.* (2001)^34^ to fit observations. This model allows simulating the ground deformation related to a pressurized penny-shape crack, buried within a homogeneous half-space. The degrees of freedom associated with the model are five: the three coordinates of the center of the crack, the crack radius and the overpressure. The model requires also specifying the rigidity modulus of the half-space: we selected the average value of 5 GPa^21^.

The non-linear optimization process aims at minimizing the following misfit function:


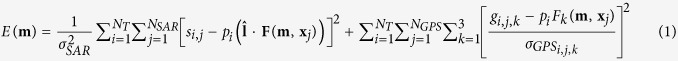


where where **m** is a four-dimensional vector representing the geometrical parameters of the Fialko *et al.* (2001) model, i.e., the three coordinates of the crack center and its radius. The index *i* spans the time intervals (N_T_ = 100) of the whole dataset, while the index *j* spans the observation points for both the SAR (N_SAR_ = 12346) and GPS (N_GPS_ = 15). SAR data are indicated as *s*_i,j_, where it is intended as the deformation value for the *j*-th pixel at the *i*-th time interval. A similar meaning holds for the GPS data, indicated as *g*_i,j,k_, with the index *k* spanning the three spatial components. The forward modeling operator **F** is a function providing the value of the ground deformation vector for a given model **m** at a specified position **x**_j_. The unit vector 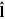 identifies the average line-of-sight direction toward the satellite position, thus allowing computing the corresponding projection of the ground deformation vector. The value of σ_SAR_ is assumed as 0.5 cm uniformly over the area^18^, while the value of σ_GPS_ depends upon the time, the sensor position and the measured component^11^, but it is usually lower than σ_SAR_.

For a given model **m** we compute the ground deformation for a unitary pressure value inside the crack. This allows determining for each time interval the best fit pressure p_i_, using a linear least square approach and then allows us to calculate the misfit function E represented in the previous expression. The minimization of the function E requires a non-linear approach: we used the Nelder-Mead simplex algorithm^35^. Furthermore, using a linearized analysis of the covariance matrix, around the best-fit model, we determined the model standard deviations and the correlation matrix (see Tables S1 and S2 in the Supplementary Material).

### Geodetic imaging

Geodetic imaging is a technique, originally developed by Vasco *et al.* (2002)^36^, aimed at retrieving the tridimensional spatial distribution of volumetric ground deformation sources by the inverting geodetic measurements. We have adapted this technique to image the opening of planar, sill-like, sources.

We consider the observed ground deformation as a representation integral between the sill opening distribution o(**y**) and the kernel Г:


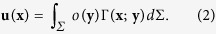


In the previous expression the surface integral is intended over a planar surface, corresponding to the sill, located at a depth of about 3100 m (Table S1). We discretized the sill using a regular grid with a cell spacing of 500 m within a radius of 4 km from the center of the caldera have been used. The discrete version of the kernel Γ was computed using the Okada (1985)^37^ model, considering each cell as a small horizontal crack. The discretized version of the previous equation lead to the classic matrix formulation of a linear inverse problem **d** = **G m**, with **d** being the observation vector (SAR + GPS), **m** the model vector (i.e. the opening for each discrete cell) and **G** the discretized kernel. To solve the problem we adopted the approach of Leao and Silva (1989)^38^, originally developed for robust downward continuation of potential fields. In this approach a generalized inverse matrix **G**^†^ is computed using the expression:


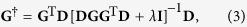


where λ is a damping parameter and the diagonal normalizing matrix **D** is defined as:


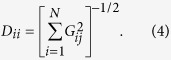


To determine the optimal value of λ we adopted the L-curve approach^39^.

**Figure 5:**
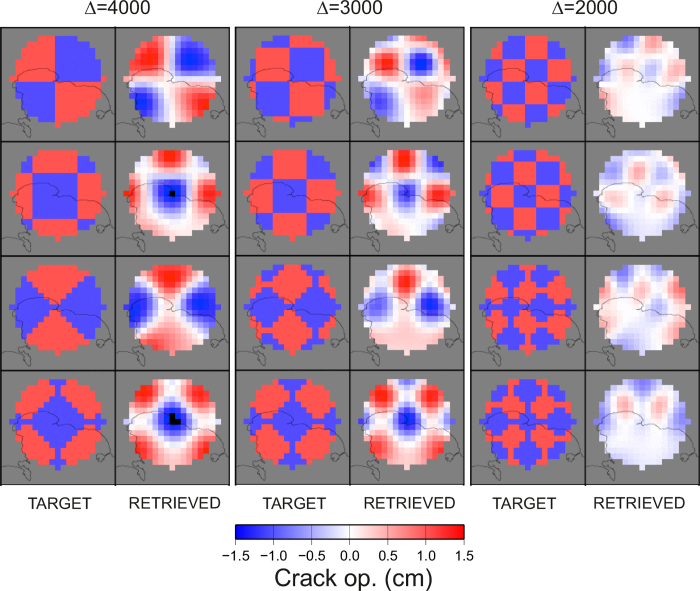
Checkerboard tests at varying wavelengths (Δ) for the geodetic imaging technique. For each wavelength we testes 4 different orientations of the checkerboard. All the maps were realized with the software GMT (http://gmt.soest.hawaii.edu).

**Figure 6:**
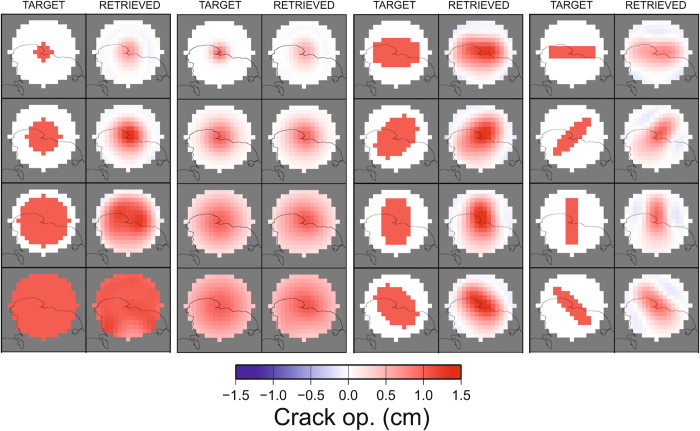
Fixed geometry tests for the geodetic imaging technique. All the maps were realized with the software GMT (http://gmt.soest.hawaii.edu).

**Figure 7:**
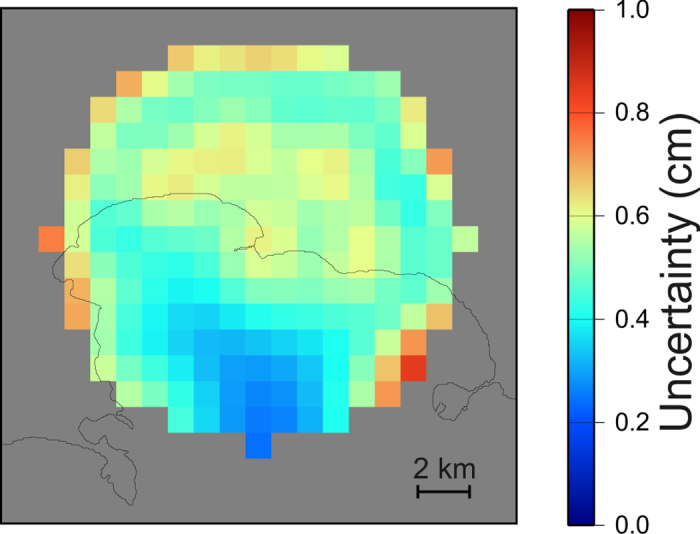
Estimated uncertainty for the geodetic imaging technique. All the maps were realized with the software GMT (http://gmt.soest.hawaii.edu).

**Figure 8:**
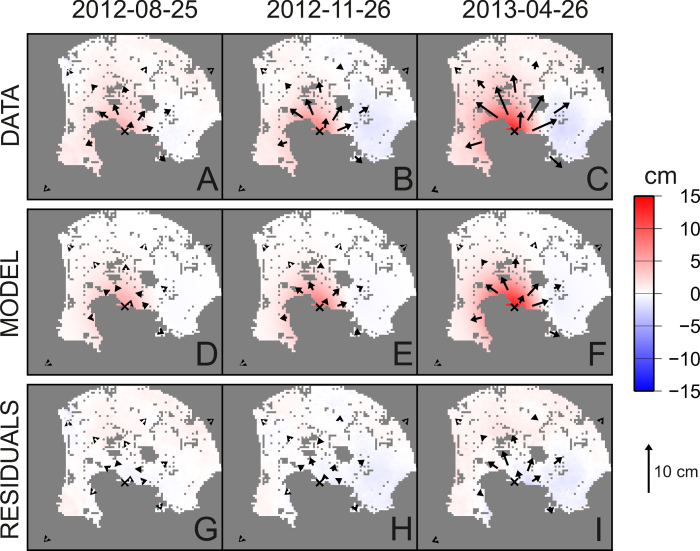
Data, model and residuals for the geodetic imaging technique for three selected intervals. (**D**–**F** in Fig. 1). (**A**–**C**) Observed deformation. The color shading represents the COSMO-SkyMed LOS displacements. Horizontal GPS displacements are indicated by arrows whose length scale is shown on the bottom right. (**D**–**F**) Synthetic model. (**G**–**I**) Corresponding residuals (data-model). All the maps were realized with the software GMT (http://gmt.soest.hawaii.edu).

Furthermore, we used canonical checkerboard (Fig. 5) and fixed-geometry tests (Fig. 6) to study the spatial resolution of the geodetic imaging results. The checkerboard tests show that the resolution of the imaging extends up to 2000 m, even if with a loss within the Pozzuoli Bay, where there is an obvious lack of measurements. Fixed geometry tests shows that, although with a smoothing of the target geometry, our approach is able to retrieve the geometry and the orientation of the sill with a good accuracy (Fig. 6). The estimated uncertainty is below 1 cm for the whole model (Fig. 7) and the fit between observations and synthetic model is good (Fig. 8).

### Inversion of the sill parameters

Using the axisymmetric model of Macedonio *et al.* (2014)^24^, we seek for the best fit parameters: η (viscosity), V^ini^ (magma already present within the sill) and ϕ(t) (magma injection rate) to fit the kinematics retrieved by geodetic imaging; note that the kinematics is expressed in terms of a function o(r, t), where o represents the crack opening, r the radial distance from the center of the sill and t is the time. For each combination of η and V^ini^ we inverted for the time-varying injection rate using a linearized approach: we start using a simple piecewise linear curve with only 3 nodes, to render ϕ(t). Subsequently, we progressively refine the representation of ϕ(t), using an increasing number of nodes (see Fig. S1 in the Supplementary material). We stop when the Akaike Information Criterion^40^ found the optimal model.

We explored all the realistic values of η and V^ini^ using a systematic grid search (Fig. 3A). For each combination of η and V^ini^, we obtained a misfit function which was used, through a probabilistic approach, to compute an “a posteriori” p.d.f. This function allowed determining the maximum likelihood model (the red dot in Fig. 3A) and bounds at a confidence level of 95% (red contour and red lines in Fig. 3A).

**Figure 9:**
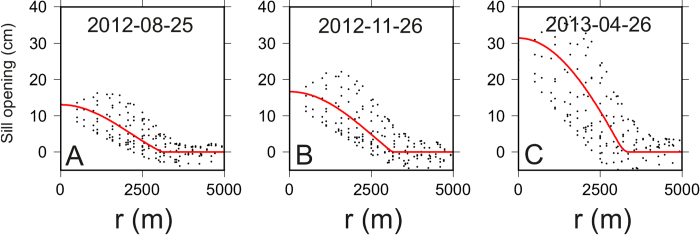
Fit between the numerical model (red line) and the retrieved sill opening kinematics (black points) for three selected intervals (D–F in Fig. 1).

In Fig. 9 we show the good agreement between the model (red line) and the sill opening kinematics.

### Finite Element Modeling of the stress field

We performed a 3D finite element modeling of the elastic stress field associated with the intrusion of a magmatic sill. We evaluated the spatial distribution of the deviatoric stress magnitude (τ_MAX_) by exploiting the geometry and the cumulative aperture of sill, in correspondence to the September 2012 seismic swarm beneath the Pozzuoli town.

We first build up the model geometry considering a domain of 22 × 16 × 10 km^3^ and using the CFc topography. Linear elastic isotropic mechanical properties were assumed, with a Young modulus of 5 GPa, a Poisson’s ratio of 0.25, and a density equal to 2500 kg/m^3^ 21. The boundary conditions of the computational domain were chosen as fixed constrain at the bottom side and roller conditions at the lateral sides of the model. We discretized the computational domain by using a mesh of tetrahedral elements whose dimension ranges from 50 to 1000 m.

In order to reproduce the lithostatic conditions inside the caldera, a pre-stress step was simulated to take into account for the gravitational loading. Subsequently, we reproduced a cumulative sill opening, with a maximum opening of 12 cm at the center, which corresponds to our modelled sill intrusion during the January-September 2012 time interval.

The comparison of the computed 3D stress field changes and the natural seismicity emphasizes a good spatial fit between the location of maximum shear stress and the spatial clustering of the events belonging to the September 7^th^ 2012 seismic swarm. The shear stress increment in the area is about 0.6 kPa (Fig. 4).

## Additional Information

**How to cite this article**: D’Auria, L. *et al.* Magma injection beneath the urban area of Naples: a new mechanism for the 2012–2013 volcanic unrest at Campi Flegrei caldera. *Sci. Rep.* **5**, 13100; doi: 10.1038/srep13100 (2015).

## Supplementary Material

Supplementary material
