# Supplementary material for: Magma injection beneath the urban area of Naples: a new mechanism for the 2012–2013 volcanic unrest at Campi Flegrei caldera
Source: Sci Rep. 2015 Aug 17;5:13100. doi: 10.1038/srep13100 (PMC4538569; doi:10.1038/srep13100)
Supplement: Supplementary material [file srep13100-s1.doc]

# Supplementary material

# Magma injection beneath the urban area of Naples: a new mechanism for the 2012-2013 volcanic unrest at Campi Flegrei caldera.

*Luca D'Auria^1,2*^, Susi Pepe^2^, Raffaele Castaldo^2^, Flora Giudicepietro^1^, Giovanni Macedonio^1^, Patrizia Ricciolino^1^, Pietro Tizzani^2^, Francesco Casu^2^, Riccardo Lanari^2^, Mariarosaria Manzo^2^, Marcello Martini^1^, Eugenio Sansosti^2^, Ivana Zinno^2^*

^1^Istituto Nazionale di Geofisica e Vulcanologia, sezione di Napoli Osservatorio Vesuviano, via Diocleziano 328, 80124 Napoli (Italy).

^2^National Research Council of Italy (CNR), Istituto per il Rilevamento Elettromagnetico dell’Ambiente, via Diocleziano 328, 80124 Napoli (Italy).

*Correspondence to: luca.dauria@ingv.it

| **Parameter** | **Description** | **Value** |
| --- | --- | --- |
| X_c_ | X coordinate of the crack center | 280±29 m |
| Y_c_ | Y coordinate of the crack center | -658±48 m |
| Z_c_ | Depth of the crack | 3090±138 m |
| R | Radius of the crack | 1089±380 m |

**Table S1**. Best fit parameters for the non-linear inversion. X_c_ and Y_c_ are relative to the point of geographic coordinates 40.82 N, 14.12 E.

|  | **X_c_** | **Y_c_** | **Z_c_** | **R** |
| --- | --- | --- | --- | --- |
| **X_c_** | 1 | 0.18796 | 0.60889 | -0.57954 |
| **Y_c_** | 0.18796 | 1 | 0.50178 | -0.55011 |
| **Z_c_** | 0.60889 | 0.50178 | 1 | -0.96354 |
| **R** | -0.57954 | -0.55011 | -0.96354 | 1 |

**Table S2**. Correlation matrix for the non-linear inversion results.


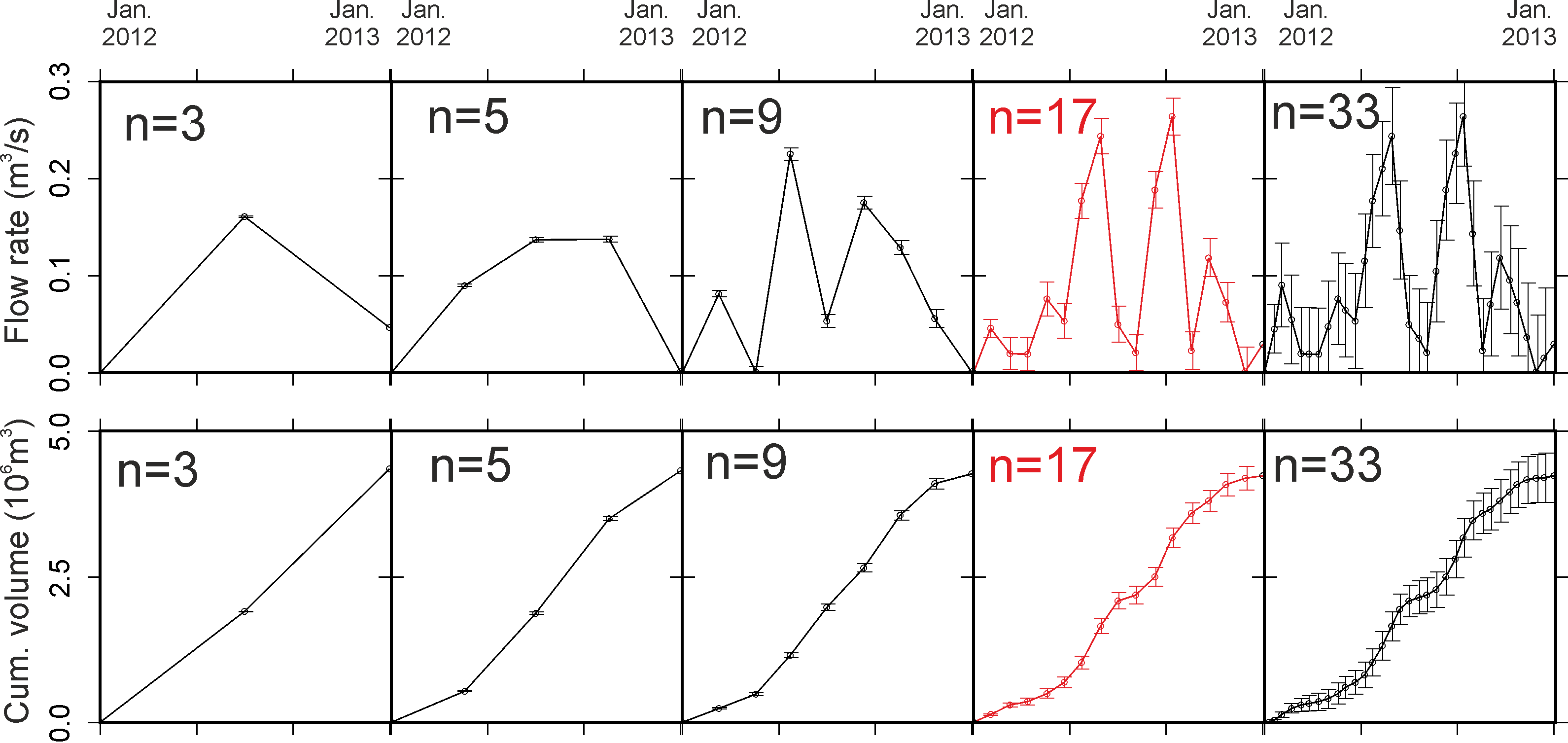


**Fig. S1.** Increasing number of nodes for the piecewise linear representation of the injection rate. On the top row we show the injection rate, while on the bottom one its cumulative curve. The red line (for 17 nodes) has been selected, using the Akaike Information Criterion.
